# ABTS/PP Decolorization Assay of Antioxidant Capacity Reaction Pathways

**DOI:** 10.3390/ijms21031131

**Published:** 2020-02-08

**Authors:** Igor R. Ilyasov, Vladimir L. Beloborodov, Irina A. Selivanova, Roman P. Terekhov

**Affiliations:** Department of Chemistry, Sechenov First Moscow State Medical University, Trubetskaya Str. 8/2, 119991 Moscow, Russia; vlbe@list.ru (V.L.B.); irinaselivanova@yandex.ru (I.A.S.); r.p.terekhov@yandex.ru (R.P.T.)

**Keywords:** ABTS, TEAC, antioxidant capacity, coupling reaction, adducts formation, flavonoids, quercetin, taxifolin

## Abstract

The 2,2′-azino-bis(3-ethylbenzothiazoline-6-sulfonic acid) (ABTS^•+^) radical cation-based assays are among the most abundant antioxidant capacity assays, together with the 2,2-diphenyl-1-picrylhydrazyl (DPPH) radical-based assays according to the Scopus citation rates. The main objective of this review was to elucidate the reaction pathways that underlie the ABTS/potassium persulfate decolorization assay of antioxidant capacity. Comparative analysis of the literature data showed that there are two principal reaction pathways. Some antioxidants, at least of phenolic nature, can form coupling adducts with ABTS^•+^, whereas others can undergo oxidation without coupling, thus the coupling is a specific reaction for certain antioxidants. These coupling adducts can undergo further oxidative degradation, leading to hydrazindyilidene-like and/or imine-like adducts with 3-ethyl-2-oxo-1,3-benzothiazoline-6-sulfonate and 3-ethyl-2-imino-1,3-benzothiazoline-6-sulfonate as marker compounds, respectively. The extent to which the coupling reaction contributes to the total antioxidant capacity, as well as the specificity and relevance of oxidation products, requires further in-depth elucidation. Undoubtedly, there are questions as to the overall application of this assay and this review adds to them, as specific reactions such as coupling might bias a comparison between antioxidants. Nevertheless, ABTS-based assays can still be recommended with certain reservations, particularly for tracking changes in the same antioxidant system during storage and processing.

## 1. Introduction

It has been more than two decades since one of the most widely used methods of antioxidant capacity measurement, the improved trolox equivalent antioxidant capacity (TEAC) assay, was invented [1]. This assay applies the radical cation ABTS^•+^ (2,2′-azino-bis(3-ethylbenzothiazoline-6-sulfonic acid) as a model radical and differs from the initial version of TEAC assay in a radical-initiator—potassium persulfate (PP) instead of metmyoglobin/H_2_O_2_ [2]. A recently published comprehensive overview of ABTS/TEAC assays gives an overall insight into ABTS^•+^ radical cation-based antioxidant capacity approaches, including different methods of ABTS^•+^ generation, quantification strategies, and experimental design, among others, as well as a considerable TEAC value data collection obtained using the ABTS^•+^ chromogen [3]. It can be assuredly stated that the ABTS/PP assay measures only the antioxidant capacity, does not estimate the antioxidant reactivity or concurrent inhibition rates, and it has some shortcomings, which are out of the scope of this work and are comprehensively enlightened in the recent reviews, for instance, Cano et al. [3], Schaich et al. [4], or Apak [5]. This review focuses on another issue, namely, on what happens with ABTS^•+^, as well as with antioxidants, as a result of their interaction. Thus, the main objective of this review was to elucidate the pathways of ABTS^•+^ with antioxidants reactions. Due to many different TEAC assay modifications, widespread use of Trolox as an equivalent standard compound, and the TEAC index as a measure for antioxidant capacity expression in different assays, we here use the abbreviation “ABTS/PP” for the improved TEAC assay.

## 2. ABTS/PP Abundance Statistics

Despite the recent numerous reviews on the measurement of antioxidant activity/antioxidant capacity (AOA/AOC) [6,7,8,9,10,11,12,13,14] and even the emergence of a systematizing, comprehensive, and fundamental book devoted to this issue [15], the choice of a method for the study of AOA/AOC remains difficult, as none of the developed methods are recognized as universal. However, we can still say that at the moment the circle of the most widely used in vitro AOA/AOC methods is outlined, among which the ABTS/PP method has a special place (Table 1). According to the Scopus citation indexes, ABTS-based antioxidant capacity assays are among the three most popular methods, together with 2,2-diphenyl-1-picrylhydrazyl (DPPH)-based and ferric-reducing antioxidant power (FRAP). All three are commonly accepted and routinely practiced in research laboratories throughout the world.

**Figure 1:**
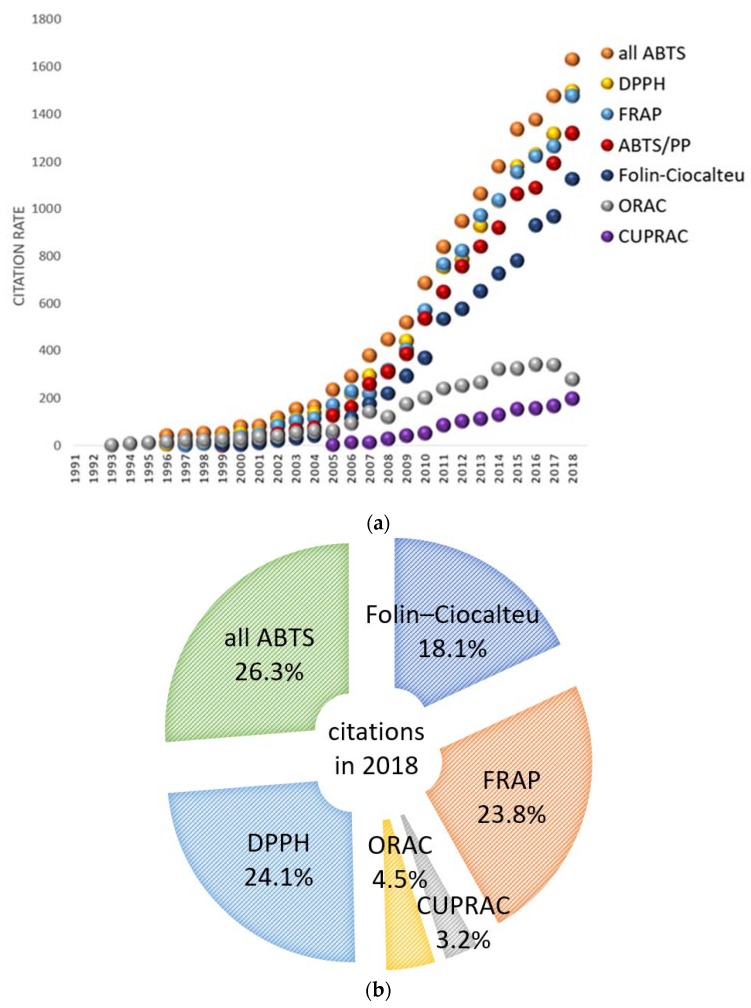
Citation rates of basic publications of the most widely used AOA/AOC methods from Table 1: (**a**) Dynamics of citation rates change by year. All methods showed a gradual increase of citations up to 2018, except for the oxygen radical absorbance capacity (ORAC) assays, whose citation number decreased in 2018 to the level of 2013. (**b**) Distribution of basic publications citations in 2018; the sum of these citations (6212 cites) was taken as 100%. Data are from the Scopus database.

The downsides of these assays have been thoroughly and constantly discussed in the literature; nevertheless, they are rather cheap, experimentally and instrumentally easy to apply, and they give fast, reproducible data. As a result, these methods are widespread, and thus researchers have an opportunity to compare their own findings with others for the same assay. We analyzed citing data based on the Scopus information source, which is a very large abstract and citation database of peer-reviewed literature. Citation index data and frequency of abbreviation use are shown in Table 1. Figure 1a gives insight into the overall picture of the method popularity and abundance considering the above-mentioned reservations. Out of the many modifications of ABTS-based assays, the ABTS/PP is the most popular, for instance, in 2018, ABTS/PP was used in approximately 80% of all ABTS-based assays citation metrics (Figure 1b).

Even though the data in Table 1 are based only on the Scopus citation index and apparently give biased data on method usage frequency due to many reasons, the high citation numbers give an opportunity to overview the general tendency.

## 3. ABTS/PP Basic Chemistry

**Figure 2:**
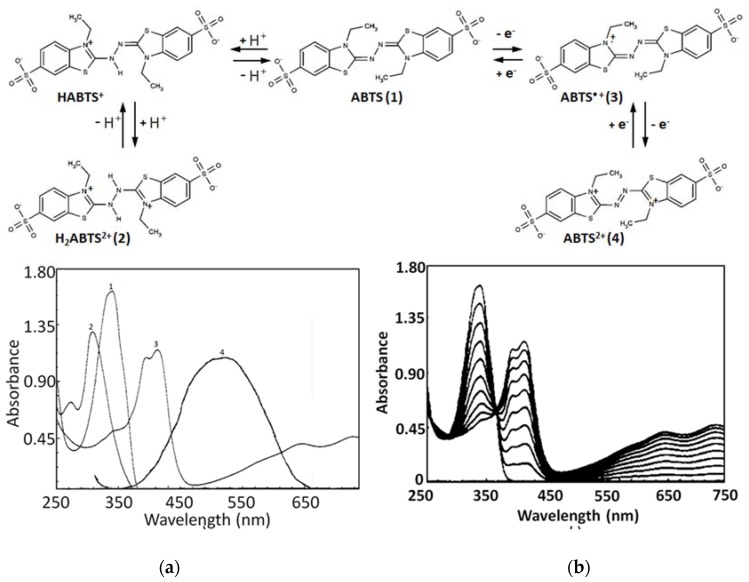
Structures of ABTS, the protonated and oxidized forms, and their absorption spectra in water: (**a**) ABTS pH 7 (1), ABTS pH 1 (2), ABTS^•+^ radical cation (3), and ABTS^2+^ dication (4); (**b**) the kinetics of ABTS to ABTS^•+^ conversion with a radical initiator. Spectra are adopted from Venkatasubramanian et al. (1989) and Scott et al. (1993) [41,42]. Copyright 1994, reproduced with permission from John Wiley and Sons, and copyright 1993, reproduced with permission from the American Chemical Society, respectively.

The basis of the ABTS/PP assay is the interaction between an antioxidant and the pre-generated ABTS^•+^ radical cation. ABTS^•+^ scavenging can be easily quantitatively detected due to the bleaching of absorption spectrum characteristic maxima at 414, 417, 645, 734, and 815 nm (Figure 2a). The usually applied characteristic maxima to monitor are 414–417 nm and 730–734 nm (Table 2); however, the latter is the recommended range due to the possible interference of many samples, which can be expected at lower wavelengths, resulting in the underestimated antioxidant capacity [37]. It is important to consider that ABTS^•+^ absorbance maximum bands shift a little in different solvents due to the solvatochromic effect: methanol (744–745 nm), ethanol (753 nm), and propanol-1 (757 nm) [38,39]. The pH impacts the λ_max_ either, for example, in 0.1 M acetate buffer pH 5 a hypsochromic shift is observed with a maximum at 728 nm [40]. The commonly used end-points for ABTS^•+^ loss detection are 4 or 6 min. Usually, ABTS^•+^ is pre-generated a day before, by mixing the PP and ABTS to stand overnight (for 12–16 h)—PP stoichiometrically oxidizes ABTS (ABTS/PP ratio 2:1, Figure 2b) to form ABTS^•+^, which can be easily observed by a color change from almost colorless to deep bluish-green. The ABTS to ABTS^•+^ conversion degree under these conditions is approximately 60%.

According to the absorption spectrum (see Figure 2a), ABTS is expected to be colorless; however, the ABTS substance is usually pale greenish. This is apparently due to trace amounts of ABTS impurities. This can be indirectly evidenced by the fact that after there is a dissolution of native ABTS in water, the received pale greenish solution immediately becomes colorless after the addition of ascorbate or any other antioxidant compound.

The formation of ABTS^•+^ apparently occurs according to the mechanism below and corresponds to a simple bimolecular reaction having a low activation energy of approximately 8 kcal/mol [41,46]:

ABTS + S_2_O_8_^2−^ ⟶ ABTS^•+^ + SO_4_^•−^ + SO_4_^2−^ (1)

ABTS + SO_4_^•−^ ⟶ ABTS^•+^ + SO_4_^2−^ (2)

The variation of pH in the range 2.0–10.5 does not affect the reaction of ABTS oxidation by PP. In the acidic medium (at a pH less than 2.0) the reaction rate decreases presumably due to the less reactive protonated forms of ABTS (see Figure 2a) [41].

The ABTS^•+^ radical cation can undergo further oxidation to form the dication ABTS^2+^ with λ_max_ at 513–520 nm, ε 36 000 L mol^−1^ cm^−1^ (see Figure 2a) [42]). The ABTS^2+^ formation kinetics from ABTS^•+^, when an excess of S_2_O_8_^2−^ is applied, is approximately three times slower than the radical cation ABTS^•+^ formation from ABTS [47,48]. Apparently, the ABTS^2+^ dication does not influence ABTS/PP assay measurements as it has rather low solubility in water and is relatively stable only in the excess of S_2_O_8_^2−^ anions or in a very acidic medium, with any aqueous dilution resulting in a shift of the reaction balance back to ABTS^•+^ [41,47,48,49,50,51].

Generally speaking, both “cation” and “dication” terms for compounds **3** and **4** are questionable as the total charge implies that they are an anion and neutral compound, as well as a dianion for ABTS itself (**1**). However, taking into account the fact that the basic work on the “improved ABTS assay” authored by Re et al. uses the “radical cation” term, and, as a consequence, that almost all authors who apply ABTS assay use this “radical cation” term, we also use it in this review.

The ABTS^•+^ is rather stable and can be used even if not prepared the night before, but one should take into account that in storage, the ABTS^•+^ gradually decomposes, and thus appropriate blanks should be recorded before each measurement; however, the decomposition rate sufficiently decreases provided that storage temperature is kept below 5 °C [3,45,46,52]. As ABTS^•+^ partly turns back to ABTS, which is evidenced by the absorbance increase at 340 nm, the ABTS^•+^/ABTS ratio changes, which can sometimes affect the measured antioxidant capacity [53,54,55,56].

## 4. Reaction Stoichiometry

ABTS^•+^ is a non-natural metastable radical, and there is general controversy of the translation of the results of simple colourimetric antioxidant assays with a physiological context, as well as the ignorance of the rate of radical trapping by antioxidants, which must necessarily be taken into account along with antioxidant capacity to reflect the true antioxidant reactivity. Another drawback of the ABTS^•+^ model is its difficult to predict chemistry. The latter leads to the complicated kinetic patterns, elevated stoichiometries, and consequent ambiguity of results and their interpretation. One of the most serious shortcomings of ABTS-based methods is that, even for some standard antioxidants such as quercetin, naringin, or glutathione, different TEAC values can be observed between laboratories. The main two reasons are the dependence of the relative antioxidant capacity (e.g., TEAC index) on the concentration for some antioxidants [52,57,58,59] and the incompleteness of ABTS^•+^ scavenging reaction by 6th and even more so by the 4th minute; moreover, by the 30th min some antioxidants still demonstrate gradual ABTS^•+^ inhibition [56,60,61,62,63,64,65].

For this reason, even if one measures ABTS^•+^ scavenging in the 20–60% range, the obtained results are going to differ from the same samples measured in the 30–70% range or taken as IC_50_. Therefore, results measured at the fourth min vary from those at the sixth min or later, and a quite common picture is the lifted curve of the *∆A_ABTS_^•+^ = kC_antioxidant_+b* equation at a small concentration area with a relatively high *b* coefficient, far from being equal to zero. One of the reasons for such behavior can be attributed to the critical role of the steric accessibility to the ABTS^•+^ radical site [57]; another is the occurrence of sideline reactions such as coupling adduct formation and their own ABTS^•+^ radical scavenging reactivity.

**Figure 3:**
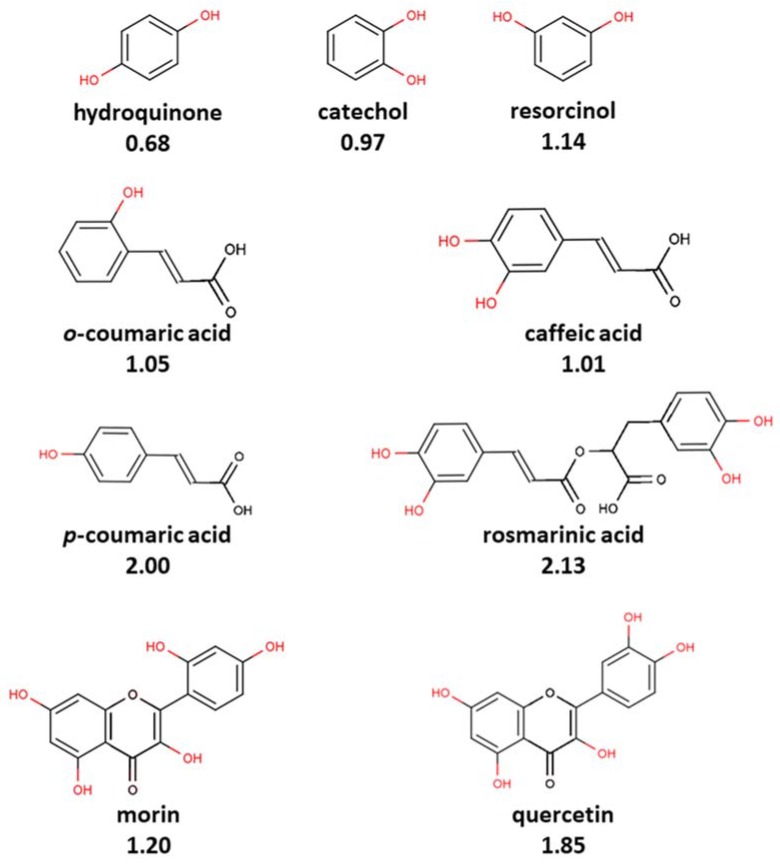
The number of hydroxyls does not always correlate with the TEAC value in ABTS/PP assay [71]. Hydroquinone, catechol, and resorcinol have the same number of hydroxyl groups, but there is a sufficient difference in the TEAC value, as well as in case of morin and quercetin. However, p-coumaric acid and rosmarinic acid showed the same TEAC-value, as well as o-coumaric acid and caffeic acid, in spite of twofold and fourfold differences in the number of hydroxyl groups, respectively. The TEAC values in ethanol are given under the name of each compound.

The stoichiometry of the process is another serious concern. The glutathione (GSH) was found to give higher scavenging stoichiometry with ABTS^•+^ than the expected 1-eoxidation, which was possibly due to the deeper GSH oxidation to sulphenic and sulphinic acids rather than only to glutathione disulfide glutathione disulfide (GSSG) [58,66]. This was evidenced by the fact that oxidized glutathione GSSG also showed the ability to slowly decolorize ABTS^•+^, apparently due to the further oxidation of sulfur [60,67,68]. Another illustration is the number of ABTS^•+^ scavenged after 10 min of incubation, which was about 5.0 for tryptophan or 4.0 for tertbutylphenol; stoichiometric factors are difficult to reason and provoke doubts on the validity of ABTS^•+^ for the antioxidant capacity determination [46]. The number of phenolic hydroxyl groups generally correlated with the ABTS^•+^ scavenging capacity [69,70]; however, some results are out of this line (Figure 3).

For instance, *p*-coumaric (one phenolic OH group) acid was almost equally active to rosmarinic acid (four OHs), and *o*- and *m*-coumaric and isoferulic acids (one OH each) were almost equal in activity to caffeic acid (two OHs); resorcinol and phloroglucin, which have meta-substitution pattern of OH-groups, were more active than catechol and hydroquinone, having ortho- and paradihydroxy substitutions, respectively [1,71]. Moreover, the stoichiometry for flavonoids is peculiarly high, for example, the quercetin/ABTS^•+^ ratio observed was 1:12, and morin, whose only difference from quercetin is the position of one hydroxyl in the B-ring (2′ in morin instead of 3′ in quercetin), showed almost twice the stoichiometry (Figure 3 and Table 3). There are many more examples that are out of the scope of this review; however, all of them witness the difficulty of predicting the chemistry of the ABTS/PP assay.

To get more insight into the chemistry of ABTS^•+^ scavenging, it is essential to consider the system modelled (Table 4). In the case of the ABTS/PP decolorization assay, there were three major components in the reaction medium: pre-generated ABTS^•+^, antioxidant, and the non-reacted and reduced form after interaction with antioxidant ABTS. The ABTS^•+^ concentration, as well as the ABTS concentration (around 47 and 31 μmol/L, respectively), was sufficiently higher than the antioxidant concentration (see Table 4). For instance, to inhibit 50% of ABTS^•+^, even in the case of such a rather weak antioxidant as Trolox (around IC_50_ 11 μmol/L), the Trolox/ABTS^•+^ ratio was approximately 1:4. However, when a strong antioxidant such as quercetin was added to inhibit 50% of ABTS^•+^, its concentration around 2 μmol/L was 25-fold less than that of ABTS^•+^.

The consequence of this considerable excess of ABTS^•+^ was that the total equilibrium of the ABTS^•+^ scavenging reaction shifted to the right as much as possible, pushing the antioxidant to show maximum reactivity. All intermediates or covalent adducts that have their own AOA were also likely to react with the ABTS^•+^ radical giving such high stoichiometric coefficients (see Table 3). It is likely that this excess of ABTS^•+^, as well as the relatively high rates of ABTS^•+^ consumption in sideline reactions, are among the reasons for the high stoichiometry. For example, the same ABTS/PP system gave much more adequate stoichiometric coefficients when ABTS^•+^ was generated in situ in the presence of antioxidants in a lag-time assay [58]. In this variant, the solution simultaneously contained antioxidant, potassium persulfate, and the excess of reduced/unreacted ABTS.

As for the ABTS^•+^ radicals, the main difference of the lag-time assay from the decolorization assay was that their concentration during the lag-time was close to zero due to the capture of the generated radical cations by antioxidants, and it began to grow only after the consumption of most of the antioxidant. Finally, there was no great excess of ABTS^•+^ and, as a result, the stoichiometries for most antioxidants were close to the real-life reasonable ranges. For example, glutathione in the decolorization assay scavenged approximately three molecules of ABTS^•+^, whereas the lag-time assay gave a physiologically plausible stoichiometric factor close to 1 (see Table 3). Apparently, due to the gradual inflow of ABTS^•+^, only the fastest reaction prevailed, that is, ABTS^•+^ scavenging, and not the coupling or adducts formation and these products’ consequent self-scavenging of ABTS^•+^.

At the same time, naringenin and apigenin did not give any classical initial lag-time at all—ABTS^•+^ accumulation in their presence showed different kinetics, demonstrating no initial lag-time, but a kind of delayed lag-time. Finally, morin scavenged ABTS^•+^ in an easily distinguishable two-stage manner, giving both the initial and delayed lag-time [58].

These kinetic patterns together with the biphasic or more complicated kinetics revealed in other reports [57,60,72,73,74] prove the fact that not only the parent antioxidant but also its intermediates do scavenge ABTS^•+^ radicals, contributing to the overall antioxidant capacity. Moreover, it appears that intermediates can demonstrate even higher kinetic rates, as it is outlined for naringenin or apigenin and greater antioxidant capacity. Arts et al. performed elucidation of reagents and product concentration dynamic changes for the chrysin or trolox reaction with ABTS^•+^ by means of high performance liquid chromatography with ultraviolet detection (HPLC-UV) [64]. It was clearly demonstrated that chrysin totally consumed 5.8 molecules of ABTS^•+^, out of which only 30% could be attributed to the chrysin self-scavenging activity, and the remaining 70% were suggested to be scavenged by reaction product(s); furthermore, the reaction rate of the product with ABTS^•+^ was deduced to considerably exceed that of chrysin with ABTS^•+^. In contrast, Trolox formed a non-reactive reaction product after direct scavenging of about two molecules of ABTS^•+^.

## 5. Reaction Pathways with Adducts Formation

The most common suggestion with concern to ABTS^•+^ is that an antioxidant reduces it to the parent substrate ABTS.

**Figure 4:**
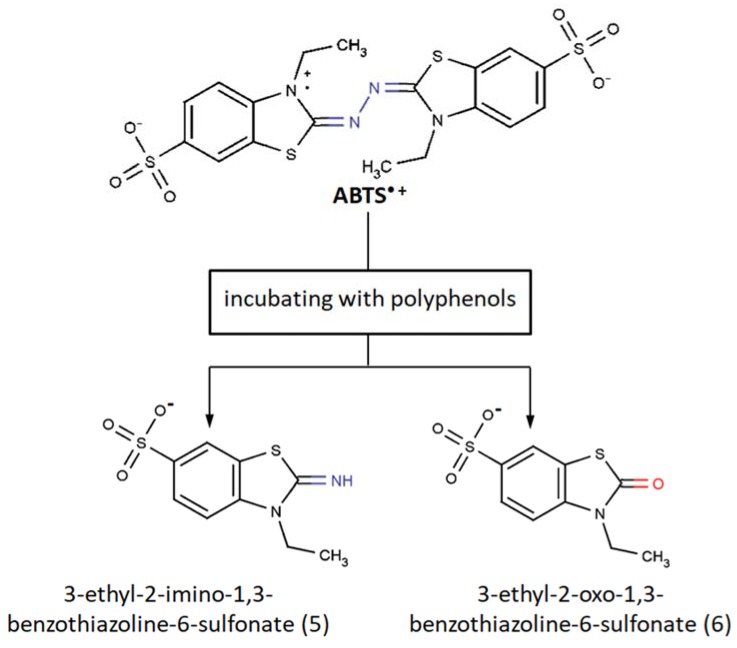
Products of ABTS^•+^ degradation [75].

In contrast, Osman et al. reported that ABTS at least partly degraded as a result of reactions with polyphenols [75]. Two products of ABTS^•+^ cleavage were formed when incubating with catechin, epicatechin, or phloroglucinol, whereas the formation of the degradation products was negligible in the absence of the polyphenols and no product formation was observed in the absence of the ABTS^•+^, indicating that the polyphenols themselves were stable under that experimental conditions. These two products were isolated, purified by preparative HPLC, and subsequently characterized with UV-VIS, ^1^H NMR, analysis of the elemental composition, and mass-spectrometry. Two structures were proposed, namely, 3-ethyl-2-imino-1,3-benzothiazoline-6-sulfonate (**5**) and 3-ethyl-2-oxo-1,3-benzothiazoline-6-sulfonate (**6**) (Figure 4).

Apparently, these products result from oxidative cleavage of the two nitrogen-linked benzothiazole rings. Unfortunately, there is a lack of quantitative estimation of these products’ formation except that authors have carefully mentioned that only around 20% of the original amount of the substrate ABTS was collected. Three additional points should be taken into account with regard to this report:

(1) As ABTS^•+^ was generated in the ABTS/laccase system, but not ABTS/PP, one could argue that it could hypothetically influence the observed ABTS^•+^-antioxidant reaction and subsequent ABTS^•+^ depletion. There are many precedents when certain antioxidants reveal different antioxidant capacities against the same model radical but a different radical-generating system, for example in the ABTS/metmyoglobin/H_2_O_2_ assay, the TEAC of quercetin and cyanidin was 4.72 and 4.4 [76,77], whereas in the ABTS/PP assay it was 3.03 and 2.48 [1], respectively. Additionally, laccase can act as an oxidizing agent alone, not to mention that the combination of laccase with ABTS or other mediators shows a higher oxidation ability than laccase or ABTS^•+^ separately [78,79,80,81,82]. Nevertheless, these concerns seem to be unfounded, as the same researchers observed these ABTS^•+^ degradation products again in their next report when applying the ABTS/PP system [83].
(2) The time of reaction was 1–2 h, which is much more than the 4–6 min in the original TEAC assay, and thus there was much more time for ABTS^•+^ cleavage.
(3) The fact that polyphenol was added “dropwise” to the concentrated ABTS/laccase mixture, which also differs from the original design of ABTS/PP assay, did not seem to matter either, as generally the same situation C(ABTS^•+^) > C(antioxidant) at any given time was reproduced.

**Figure 5:**
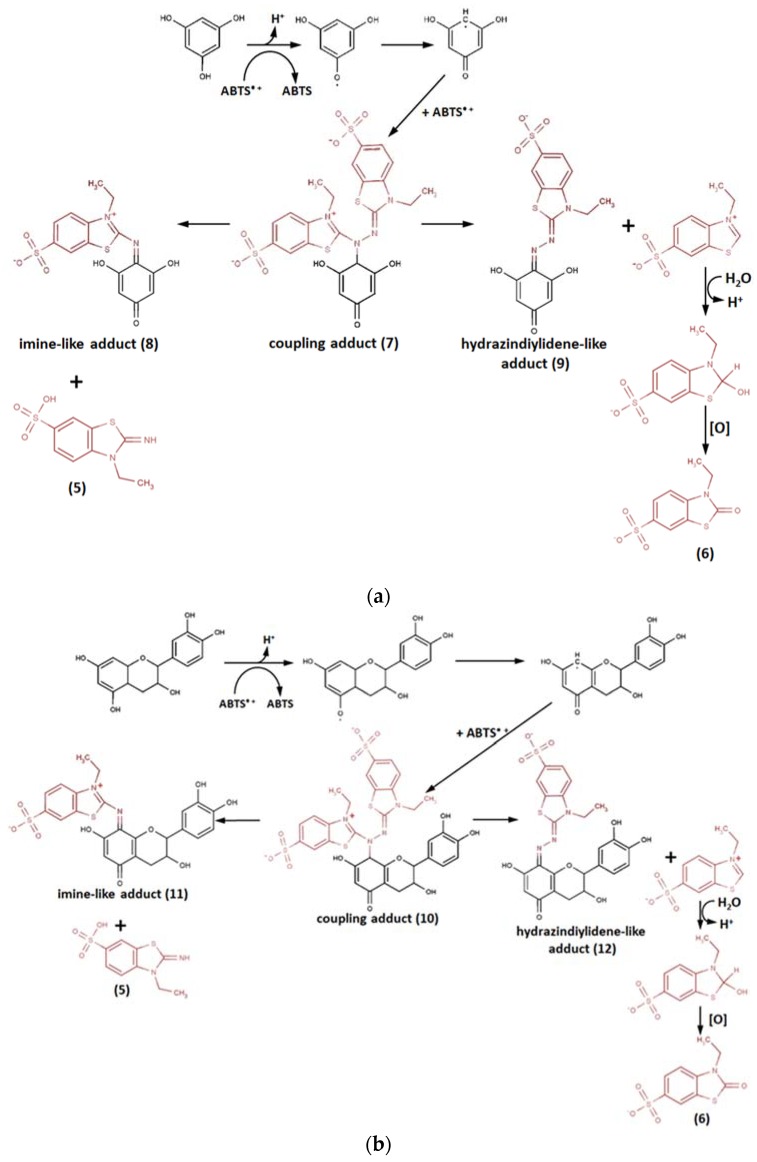
Reaction pathways of ABTS^•+^ with (**a**) phloroglucinol and (**b**) catechin [83]. Copyright 2006, adapted with permission from Elsevier.

Further findings showed that both ABTS^•+^ degradation products were supposedly not the consequence of self-cleavage, but the result of subsequent adduct formation and further degradation (Figure 5) [83].

Preparative HPLC followed by electrospray ionization mass spectrometry (ESI-MS) analysis of the products of phloroglucinol with ABTS^•+^ radical cation reaction suggested the formation of two major compounds (Figure 5a): covalent phloroglucinol-ABTS coupling adduct (**7**) having a m/z value of 639.6 (PI) and its hydrazinediylidene-like derivative (**9**) m/z 396.1, as well as the minor imine-like adduct (8) with m/z 381.3. Two ions with m/z 259 and 259.1 assigned to ABTS^•+^ degradation products were detected as well. The two adducts of the catechin/ABTS^•+^ reaction had m/z values of 803 and 560, the coupling reaction product (**10**) and hydrazinediylidene-like derivative (**12**), respectively (Figure 5b). Small amounts of the imine-like adduct (**11**) were also presumably detected on the preparative HPLC chromatogram, but its quantity was not enough for characterization. The m/z value difference between two major polyphenol-derived adducts was the same in both cases, amounting to around 243, which can be attributed to benzothiazolium ion, the predecessor of 3-ethyl-2-oxo-1,3-benzothiazoline-6-sulfonate (**6**), the second of the previously identified ABTS^•+^ degradation products, whose formation is, likely, the indirect testimony of hydrazinediylidene-like pathway.

However, as opposed to ABTS^•+^ degradation products, these proposed structures of ABTS^•+^ adducts were not rigorously identified, as they were based only on MS/MS analysis. As for phloroglucinol, it is unlikely that other products could be guessed. In the case of catechin, it is disputable that exactly 7-OH was the first to scavenge ABTS^•+^ and, after that, the coupling underwent through the A-ring. Many structure–activity relationship studies showed much greater importance of the catechol-like B-ring of flavonoids for antioxidant activity manifestation [1,75,77,84] and the kinetic study of free-radical-scavenging action of flavonoids, and catechin in particular indicated that the activities of 5- and 7-OH groups at A-ring were rather weak and even almost negligible, whereas 3′- and 4′-OH groups at B-ring were highly reactive, suggesting that the o-dihydroxyl (catechol) structure in the B-ring is the obvious radical target site for flavonoids as well [85,86]. Finally, the examination of catechin, epicatechin, and their metabolites’ radical-scavenging potency demonstrated that a free catechol moiety in the B-ring is presumably the most important for the direct antioxidant activity and apparently leads to the formation of epicatechin quinone [87,88].

At the same time, the mechanism involved in ABTS^•+^ radical cation quenching can also contribute considerably to the domination in various reaction pathways. Several reports discuss possible mechanisms involved in ABTS^•+^ quenching, suggesting the mixed hydrogen atom transfer/single electron transfer (HAT/SET) reaction mechanism, stepwise electron transfer–proton transfer (ET-PT), and concerted electron–proton transfer (CEP) mechanism with water as the proton acceptor, among others, and apparently any of them can occur in parallel or prevail [6,9,10,55,62,89,90,91,92]. The millisecond scale reaction rates of ABTS^•+^ scavenging complicate the discrimination between possible mechanisms involved in ABTS^•+^ scavenging and the determination of the prevailing mechanism; thus, further fast flow-mix techniques are needed to elucidate the dominant mechanism [57]. Nevertheless, it is important to consider that polyphenols as the routine objects and water (commonly phosphate buffer solution (PBS) pH 7.4) or alcohols as the solvents imply the sequential proton loss electron transfer (SPLET) mechanism as the most probable.

The SPLET mechanism is as follows:

ROH ⟶ RO^−^ + H^+^ (in water or alcohols,, where ROH is, for example, phenolic comound) (3)

RO^−^ + ABTS^•+^ ⟶ RO^•^ + ABTS (4)

ABTS + H^+^ ⟶ ABTSH^+^ (5)

Due to the partial ionization of phenols in water and alcohols, phenoxide anions, which are known as being much easier and faster to oxidize than corresponding phenols, play their primary role acting as electron donors in the SPLET mechanism [14,93]. This mechanism—proposed independently by Litwinienko et al. and Foti et al. in 2004 [94,95]—seems to be the most favored for the abovementioned conditions [14,93,96]. Thus, the relative acidities of hydroxyls in catechin can determine the predominant reaction pathway.

From one point of view, the resorcinol-like A-ring is presumably slightly more acidic than the catechol-like B-ring due to the electron withdrawing nature of the hydroxyls in resorcinol, and thus the formation of an anion at 7-OH or 5-OH might seem to be preferable. In contrast, some experimental data indicate that in the case of catechin, the B-ring is slightly more acidic, even though competitive deprotonation on B- and A-rings takes place [97,98]. The comparative acidity of A- and B-rings can be critical, as flavonoid anions are better electron donors and radical scavengers than corresponding neutral molecules [99,100,101,102], whereas electron transfer at deprotonated hydroxyls prevails. Even though the pH in the reaction medium was 5.0, and less than 0.1% of catechin was ionized (taking into account the pKa_1_ of catechin around 8.7 [103,104,105]), the kinetic rates of electron transfer are normally much greater than hydrogen atom transfer, and therefore a very low anion concentration can produce a large rate enhancement and dominate [93].

Meanwhile, B-ring ortho-dihydroxyls act as electron-donating groups, decreasing O-H bond dissociation enthalpies and, hence, increasing the kinetics for hydrogen transfer [93,106]. This splitting makes it difficult to give a robust forecast for the overall effects on the direction of the reaction. Nevertheless, an A-ring adduct seems to be more reasonable according to spectra considerations of authors of this work, together with the observations made in Arnao’s group study on ABTS–flavonoid complex formation [107,108], which noted that A-ring complexes give shorter wavelength maxima than B-ring complexes.

Another point to consider is, again, the experiment design. Apart from the incubation time (2 h), which was again much longer than 4–6 min in the decolorization assay, the ratio of reagents was far from usual. In contrast to the normally observed ABTS^•+^ multiple excess in the reaction medium, the catechin/ABTS^•+^ ratio was 1:1; moreover the phloroglucinol concentration was almost 10-fold higher than ABTS^•+^ during incubation. The first consequence was that there was much more time to form adducts when the ABTS^•+^ scavenging reaction was possibly completed. The second consequence was that a lag of excess ABTS^•+^ limits the opportunity for these antioxidants to exhibit all of their antioxidant potential. ABTS-phloroglucinol adducts were tested and demonstrated their own radical scavenging activity against ABTS^•+^, thus forming further deeper oxidized forms and contributing to the total antioxidant capacity, which confirmed the prediction that chemical reactions following parent compound oxidation are at least partly in charge of their higher radical scavenging capacities [109,110].

To sum up, two points are worth mentioning:

(1) The principal possibility of ABTS^•+^ degradation and ABTS-antioxidant adduct formation was demonstrated for the first time in the ABTS/PP assay.
(2) The extent to which this degradation and adduct formation influenced the final TEAC was not obvious due to the modified experiment design and no quantitative estimations.

Controversial data were reported on catechin oxidation with the ABTS/laccase system, where no adducts were detected. Instead, dimers, trimers, tetramers, and their oxidized forms were elucidated [111]; this is another consideration in favor of the importance of the experiment design and in particular the ABTS^•+^-generating system with regard to adduct formation.

In general, the reactions of different phenols with ABTS^•+^ had been reported far before the discussed Osman et al. report and even the ABTS/PP assay invention itself. Different research groups suggested the coupling reaction between ABTS^•+^ and phenolics but without their structural elucidation, that is, ABTS^•+^ (ABTS/horseradish peroxidase/H_2_O_2_) and naringenin were reported to form the coupling product with a maximum absorption at 560 nm, whereas guaiacol and erol, the monomeric lignin model compounds, underwent coupling reactions with ABTS^•+^ (ABTS/laccase) leading to a copolymer, which could undergo deeper coupling with ABTS^•+^ to an extent that enabled their complete solubilization in water buffer, as well as several others [82,108,112,113,114]. However, the first report devoted to phenol–ABTS^•+^ coupling reaction products’ isolation and structural determination dates to 1986. Matsumura et al. studied laccase-catalyzed oxidation of 3-hydroxybenzoic and 4-hydroxybenzoic acid in the presence of ABTS as a model reaction for laccase activity or hydroxybenzoic acid concentration estimation [115,116].

**Figure 6:**
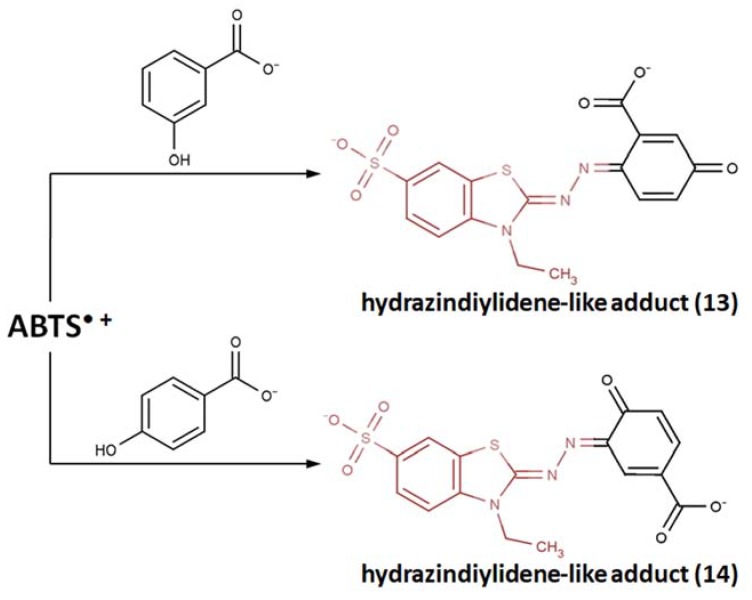
Reaction pathways of ABTS^•+^ with 3-hydroxybenzoic acid and 4-hydroxybenzoic acid [115]. Copyright 1986, adapted with permission from Taylor and Francis.

After incubation at pH 6.0, the two products were isolated via column chromatography and then thoroughly characterized and identified by means of secondary ion mass spectrometry (SIMS), UV-, IR-, ^1^H-, and ^13^C-NMR spectroscopy. Both appeared to be relatively stable hydrazinediylidene-like compounds: 6-(6’-sulfo-3’-ethylbenzothiazol-2’-ylidenehydrazono)-3-oxo-1,4-cyclohexadiene-1-carboxylate (**13**) and 3-(6’-sulfo-3’-ethylbenzothiazol-2’-ylidenehydrazono)-4-oxo-1,5’-cyclohexadiene-l-carboxylate (**14**) (Figure 6). Again, the differences between ABTS/PP and ABTS/laccase are to be considered, but in general, the same coupling pattern is difficult to argue.

**Figure 7:**
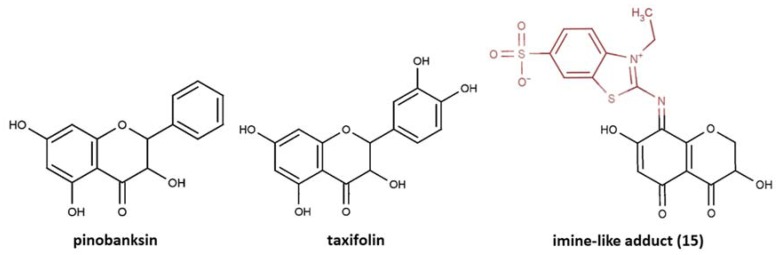
The proposed structures of ABTS^•+^ with quercetin reaction products [117].

Partially consistent results were obtained for adduct formation during the incubation of ABTS^•+^ with quercetin or its glycosides rutin, hyperoside, and quercitrin [117]. In the case of quercetin, two products were detected in the HPLC ESI-MS chromatogram along with ABTS itself. The first one was ascribed to have the potential structure of A-dihydroxy dihydroflavonol pinobanksin with m/z 319 and 301 in PI mode, which were attributed to [M+H+2Na] and [M+H−H_2_O+2Na], respectively, as well as m/z 317 [M−H+2Na] in NI mode. Another manifested as a PI ion with m/z 319.0 and 476.3, which was interpreted as the formation of dihydroflavonol-like compound taxifolin with imine-like adduct (**15**) fragmented by the elimination of ring B and 3-ethyl-2-imino-1,3-benzothiazoline-6-sulfonate (Figure 7).

Quercetin to pinobanksin and taxifolin conversions need to be evidenced by more rigorous examination, and still leave questions, such as the fact that formation of these products indicates the reduction but not the expected oxidation of quercetin, being far from the previously reported quercetin oxidation products [99,118,119,120,121,122,123,124]. For rutin, hyperoside, or quercitrin, the products of coupling with ABTS^•+^ apparently degraded with the loss of the 3-ethyl-2-imino-1,3-benzothiazoline-6-sulfonate fragment to form the imine-like adducts evidenced by the corresponding MS spectra. It is interesting to note that no hydrazinediylidene-like derivatives were revealed in this work. Whether this was due to their absence among the reaction products or whether the absorbance wavelength at 340 nm (applied for monitoring the HPLC mixtures separation) made their detection difficult, it is unclear. For instance, the previously discussed catechin-ABTS^•+^ and phloroglucinol-ABTS^•+^ major adducts appeared to be hydrazinediylidene-like derivatives, which were demonstrated to have characteristic spectra with maximum at 455 nm and almost no absorption in the 340 nm area [83]. Furthermore, the similar absorption spectra were attributed by Arnao’s group to 10 ABTS-flavonoid complexes having wavelength maxima between 445 nm and 613 nm, including quercetin (445 nm), rutin (476 nm), and hyperoside (477 nm) [107], which could make them difficult to be detected at 340 nm. Nevertheless, in the latter work, the authors pointed out that the reaction between ABTS^•+^ in the presence of horseradish peroxidase/H_2_O_2_ and flavonoid was different to that which occurred when the pre-generated ABTS^•+^ was used for antioxidant activity measurement [1,46,125,126,127]. Indeed, as we have mentioned before, another ABTS^•+^-generating system, ABTS/laccase, was revealed as a more powerful oxidant than ABTS^•+^ itself.

**Figure 8:**
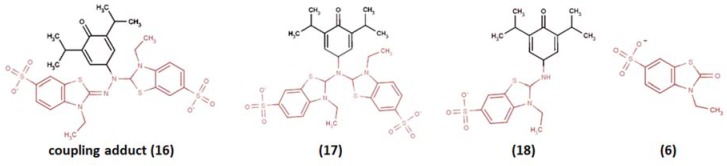
The proposed structures of ABTS^•+^ with propofol reaction products [128].

To a certain extent, a similar situation was observed for the interaction between propofol (2,6-diisopropylphenol) and ABTS^•+^ [128], which was studied in conditions reproducing Osman et al. [83] with minor modifications, wherein ABTS/PP was used for ABTS^•+^ production, the propofol/ABTS^•+^ ratio was close to 2:1, and the incubation time was 30 min. Four products were identified by means of HPLC ESI-MS in PI mode with m/z 691.5, 679.8, 437.4, and 259.5. The proposed structures (**16**) and (**18**) reproduced an imine-like reaction pathway with the reduced imine-like adduct final formation (**18**) (Figure 8). The proposed (**17**) structure formation was difficult to reason, however, and any other variant of interpretation is also far from being unambiguous as m/z 679.8 indicated the loss of only 12 mass units from the basic propofol/ABTS^•+^ coupling product with m/z 691.5. As for hydrazinediylidene-like adducts, product (**6**) indirectly witnessed their formation, but they themselves were not detected. However, in this case, the arguments about the suitability of the chosen wavelength of HPLC-UV detection does not seem reasonable enough (254 nm).

The coupling of ABTS^•+^ with another monophenol compound, the glucoside of hydroquinone arbutin, was studied by Tai et al. [129]. The formation of a purple-colored adduct was observed for the incubation of arbutin with ABTS^•+^ at a molar ratio 1:4 (pH 6, room temperature). The reaction products were thoroughly isolated on chromatographic columns and thereafter fully characterized and identified by high resolution electrospray ionization mass spectrometry (ESI-HRMS) and several NMR spectra (^1^H, ^13^C, ^1^H-^1^H COSY, heteronuclear single quantum coherence (HSQC), and heteronuclear multiple bond correlation (HMBC)).

**Figure 9:**
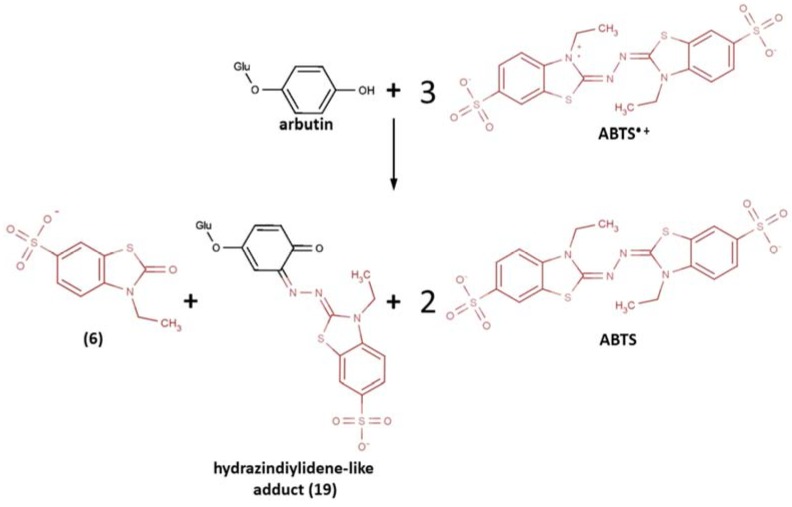
The reaction of ABTS^•+^ with arbutin [129]. Copyright 2016, adopted with permission from American Chemical Society.

Here, hydrazinediylidene-like adduct (**19**) was determined to be the major product, as well as 3-ethyl-2-oxo-1,3-benzothiazoline-6-sulfonate (**6**), whereas no imine-like adducts were detected (Figure 9). The incubation for 15 min and 120 min showed almost no difference in reaction products; moreover, the further experiments demonstrated that even 5 min was enough for the arbutin−ABTS^•+^ degradation fragment adduct formation, thus showing that the standard 6-10 min of incubation in ABTS/PP assay can be quite enough for adduct formation to contribute to the total antioxidant capacity measured. Interestingly, that arbutin−ABTS^•+^ hydrazinediylidene-like adduct (**19**) revealed no antioxidant activity in contrast to 3-ethyl-2-oxo-1,3-benzothiazoline-6-sulfonate (**6**), which revealed radical-scavenging activity, though was almost negligible.

**Figure 10:**
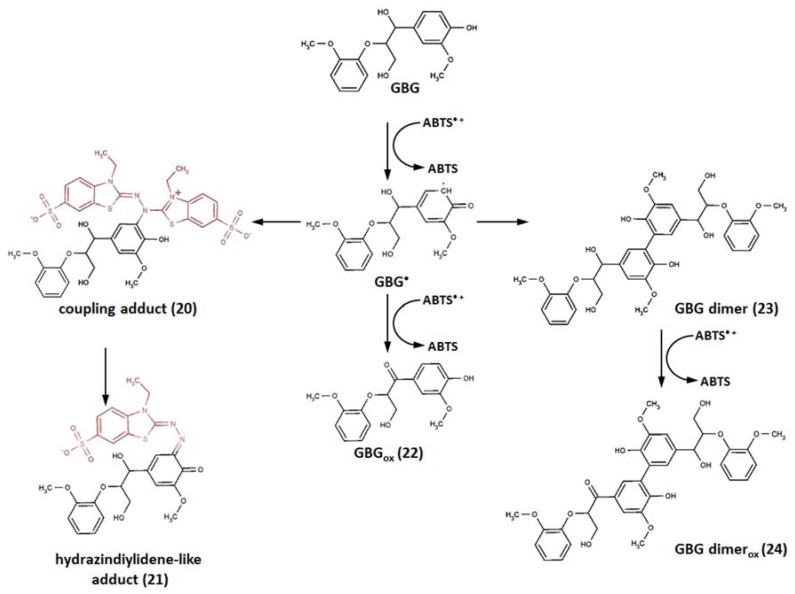
Reaction pathways of ABTS^•+^ with guaiacylglycerol-β-guaiacyl ether (GBG) [130]. Copyright 2018, adopted with permission from American Chemical Society.

The model compound mimicking the phenolic subunits in lignin, 1-(4-hydroxy-3-methoxyphenyl)-2-(2-methoxyphenoxy) propane-1,3-diol (guaiacylglycerol-β-guaiacyl ether, GBG), reported by Hilgers at al., was also able to couple with ABTS^•+^ (Figure 10) [130]. This study set out with the aim of assessing the reactivity of different laccase/mediator systems towards phenolic lignin structures. Thus, ABTS/laccase, laccase alone, and pre-generated ABTS^•+^ radical cation were applied as oxidants to assess different GBG oxidation pathways. The results partly corroborated the findings of the aforementioned reports—the ABTS^•+^, obtained in reaction with laccase (pH 4, 40 °C) and then centrifuged over centrifugal filter to get rid of laccase, alone oxidized GBG to finally form the coupling product (**20**), oxidation products of GBG (GBGox) (**22**), GBG dimer (**23**), and oxidized GBG dimer (**24**), which were detected by employing reversed-phase ultrahigh-performance liquid chromatography with photodiode array and electrospray ionization mass spectrometry detection (RP-UHPLC-PDA-ESI-MS^n^).

Surprisingly, no hydrazindiylidene-like adduct was detected. This could be ascribed to the time factor, as the 2 min incubation described in this report could be too short a time for their formation; however, personal correspondence with the author of this work revealed that 2 min was quite enough, which was confirmed by the disappearance of the green color within a very short time (indicating disappearance of the radical cations). Moreover, the measurement of the reaction mixture after 60 min gave a chromatogram identical to that of the 2 min incubation. Regarding the fact that they did not observe a hydrazindiylidene-like adduct in this incubation, in personal communication with the authors via e-mail, Roelant Hilgerssuggested that the reason was that there was too little ABTS^•+^ formed to further oxidize the coupling product to a hydrazindiylidene-like product; however, the incubation time was definitely not the limiting factor. The preliminary experiments performed by the authors of this review showed that indeed different antioxidant/ABTS^•+^ ratios can lead to different products, for instance, morin forms a yellow-colored solution in the excess of antioxidant and a reddish-colored one when the excess of ABTS^•+^ is applied, which are presumably the coupling adduct and hydrazindiylidene-like adduct, respectively. Similar results were observed for several other flavonoids (data not yet published).

The pair laccase/ABTS showed higher oxidation power—GBG was oxidized at a higher rate and gave deeper oxidation products. The reactant and product abundance timeline clearly demonstrated that GBG was converted within the first minute of incubation, giving rise to GBGox (**22**) and GBGox with an ABTS coupling product similar to the GBG-ABTS coupling adduct (**21**), whose concentrations peaked and then almost totally decreased by the 20th min. The concentration of the hydrazinediylidene-like adduct (**21**) of ABTS^•+^ and GBG was also the highest after 1 min, but decreased relatively slower and dropped to about a quarter of the initial concentration by the end of the first hour. At the same time, similar to hydrazinediylidene-like adduct of ABTS^•+^ and GBGox (**21**), as well as 3-ethyl-2-oxo-1,3-benzothiazoline-6-sulfonate (**6**), concentrations progressively increased during the first hour. Conclusively, the latter two products appeared to be the ultimate products of oxidation after 24 h.

## 6. Reaction Pathways without Adduct Formation

**Figure 11:**
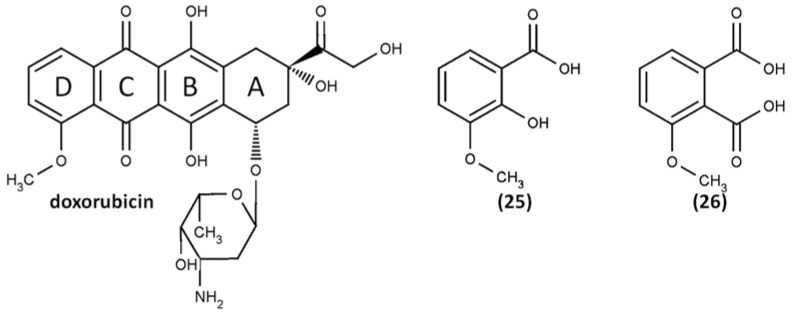
Doxorubicin and its oxidation products: 3-methoxysalicylic acid (**25**) and 3-methoxyphthalic acid (**26**) [131].

It is clear that there is not yet enough data to definitively judge the main mechanisms that underlie the reaction of ABTS^•+^ with the tested compound. In contrast to the studies outlined above that revealed coupling reaction products, there are reports that have characterized ABTS^•+^-mediated oxidation products, but with no antioxidant-ABTS adducts detected. To illustrate this fact, no adduct formation was observed in the ABTS^•+^-mediated oxidation of doxorubicin, an antineoplastic agent with anthracycline glycoside structure [131]. The oxidation products 3-methoxysalicylic acid (**25**) and 3-methoxyphthalic acid (**26**) were identified by quadrupole time of flight mass spectrometry (QTOF-MS) in comparison with authentic standards and are consistent with the literature as the expected result of doxorubicin oxidation (Figure 11) [132,133,134,135].

The ABTS^•+^ for doxorubicin oxidation was generated in situ, adding H_2_O_2_ solution to a sample consisting of ABTS and lactoperoxidase in phosphate buffer pH 7.0 giving rise to absorbances measured at 415 and 730 nm. In the presence of doxyrubicin, the same sample demonstrated no ABTS^•+^ formation for a certain period of time (lag-time), during which doxyrubicin depletion to a near zero level could be observed at 480 nm. The oxidation products for MS analysis were collected right after the lag-time when the oxidation of doxyrubicin was nearly completed (absorbance at 480 nm reached minimum). Generally, these conditions, that is, in situ ABTS^•+^ generating in the presence of H_2_O_2_, the overall exposition time (10–20 min) was found to be similar to the conditions of flavonoid-ABTS^•+^ coupling reported by Arnao’s group [107,108] and therefore adduct formation could be expected. However, the bulky structure of doxyrubicin, as well as the absence of appropriate sites of coupling, apparently limits adduct formation. The only probable “free” positions are located in the D-ring, which is unlikely to act in radical scavenging.

**Figure 12:**
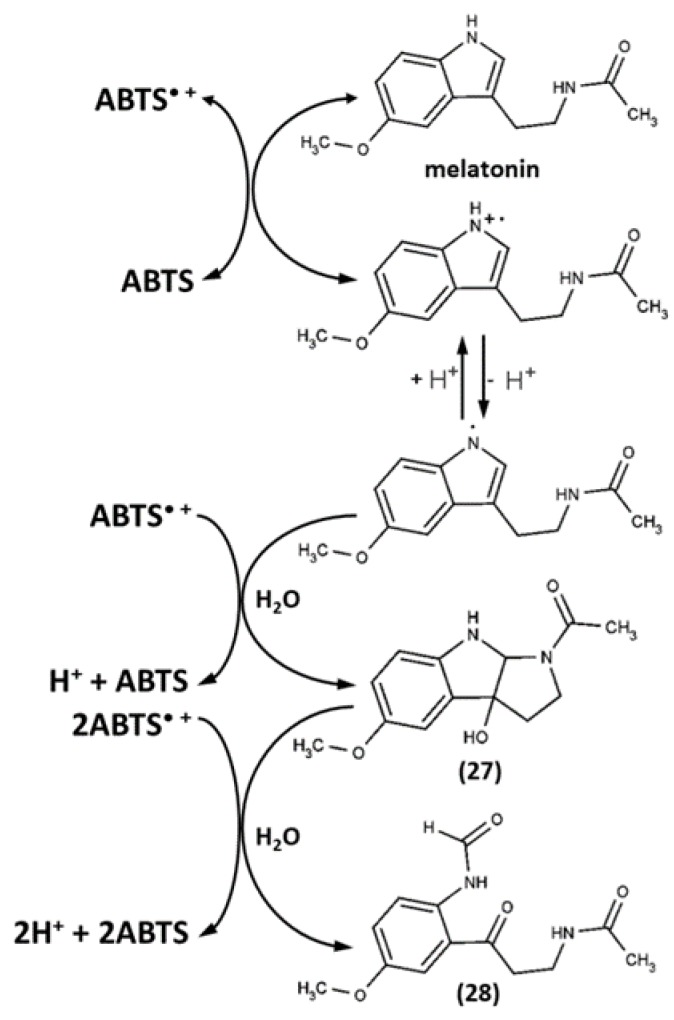
Reaction pathways of melatonin oxidation by ABTS^•+^ [136]. Copyright 2003, adopted with permission from John Wiley & Sons Ltd.

The analysis of the products of melatonin oxidation by the pre-generated ABTS^•+^ (ABTS/PP) was performed by means of high performance liquid chromatography with electrochemical detection (HPLC-ECD) with an external standard (1:1 ratio, incubation time 0.5–12.0 min, pH 7.4) and thin layer chromatography (TLC; different ratios, incubation time 4 h, pH 7.4). Two principal products were revealed: cyclic 3-hydroxymelatonin (c3OHM, 27) and *N1*-acetyl-*N2*-formyl-5-methoxykynuramine (AFMK, 28) (Figure 12) [136]. At the excess of ABTS^•+^, the dominant metabolite was AFMK; at the 1:1 ratio, c3OHM formation was threefold more than that of AFMK. Both products had been detected earlier for melatonin oxidation by other oxidants; thus, ABTS^•+^ again, as in the case of doxorubicin oxidation, gave relevant non-specific oxidation products [137,138,139]. Further examinations showed both products’ own antioxidant activity, as well as the formation of other deeper oxidation products of melatonin [59,140]. None of these studies detected any products of coupling with ABTS^•+^.

**Figure 13:**
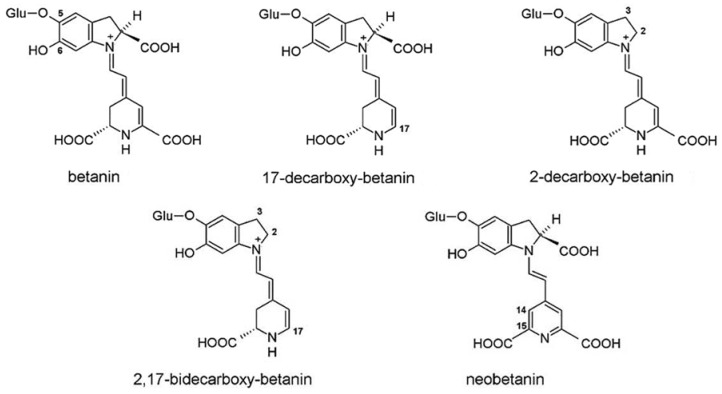
Structures of betacyanins investigated applying ABTS/PP assay [141]. Copyright 2013, adapted with permission from American Chemical Society.

The oxidation of betacyanins, namely, plant pigment betanin and its derivatives, 2-decarboxybetanin, 17-decarboxybetanin, 2,17-bidecarboxybetanin, and neobetanin, was investigated in the presence of ABTS^•+^ (ABTS/PP) cation radicals (Figure 13) [141]. The nonenzymatic oxidation mechanism was thoroughly investigated by HPLC-diode-array detection (DAD)-ESI-MS/MS.

Rather than describing all the oxidation pathways of each tested compound carefully outlined in this study, we would prefer to focus on the fact that no adduction products were detected. All the aforementioned compounds for which ABTS^•+^ adducts have been elucidated (catechin, phloroglucinol, quercetin, and its 3-O-glycosides, 2,6-diisopropylphenol, guaiacylglycerol-β-guaiacyl ether, and arbutin) are phenolics. Compounds that did not couple are doxorubicin, whose phenolic ring does not have appropriate sites of coupling, and melatonin, the non-phenolic compound. However, although betanin and its derivatives have free phenolic hydroxyl, structurally they are very close to GBG, and thus there is the question as to what the structural limitations are that interfere with ABTS^•+^ coupling. We can speculate that these are the glucoside-substitution and the only ortho to hydroxy group-free position, which together sterically hinder ABTS^•+^ attack, but undoubtedly further investigations are needed.

**Figure 14:**
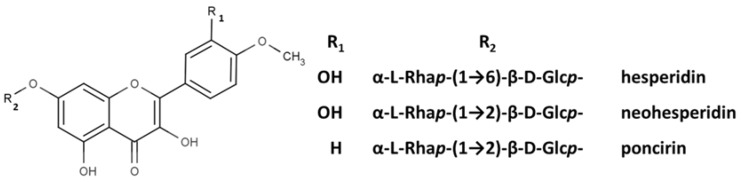
Chemical structures of hesperidin, neohesperidin, and poncirin.

Returning to Arnao’s group study devoted to ABTS^•+^-flavonoid complexes, several structural limitations were insightfully indicated [107,108]. On the basis of absorption spectra differences, they deduced that both A- and B-rings could act in complex formation, with mono- or dihydroxy substitution in the B-ring and/or 5,7-dihydroxy substitution in the A-ring favoring ABTS^•+^-flavonoid complex formation. In contrast, methylation of one hydroxyl in the B-ring, as well as 7-O-glycosylation of the A-ring, impeded their development; therefore, when these occurred together, namely, in the cases of hesperidin, neohesperidin, and poncirin, no complex formation was detected (Figure 14).

Another important point is the question of coupling adduct formation impact on ABTS^•+^-based assays in general and, particularly, the high stoichiometries obtained. Opposite to the works described above, some of which detected adduct formation almost quantitatively so that ABTS^•+^ was consumed at least partly not to regenerate ABTS, several studies reported practically complete conversion of ABTS^•+^ back to ABTS [55,142]. Is this the situation for all cases where no adducts are formed, that is, does the absence of adducts indicate that ABTS^•+^ was fully converted back to ABTS? This is another interesting issue for further elucidation.

## 7. Conclusions

Generally, the chemistry lying behind the usually oversimplified ABTS^•+^ scavenging by antioxidants is still not defined, despite the fact that the ABTS^•+^-based assays are among the most abundant antioxidant capacity assays, together with the DPPH-based assays.

Several hypotheses can be suggested concerning antioxidant-ABTS^•+^ reaction pathways for further elucidation:

(1) Some antioxidants can form adducts with ABTS^•+^, whereas others can undergo oxidation without coupling with ABTS^•+^. Thus, coupling with ABTS^•+^ is a specific reaction for certain groups of antioxidants, apparently at least of phenolic nature. Establishing the structural features that determine the direction of antioxidant interaction with ABTS^•+^ is important for future understanding and interpretation of antioxidant capacity measurements.
(2) Adduct-free oxidation pathways are substrate-specific and can be influenced both by ABTS^•+^ radical specific features or by the radical-initiator system. However, they seem to be reliably consistent with the results obtained when other oxidants are applied instead of ABTS^•+^.
(3) The coupling reaction can occur with phenolic compounds, and the coupling adduct can be the principal product as well as undergoing further oxidative degradation, which might depend on the antioxidant/ABTS^•+^ ratio and ABTS^•+^ generation methodology.
(4) Further oxidative degradation of the coupling product results from the oxidative cleavage between the two nitrogen-linked benzothiazole rings. This leads to hydrazindyilidene-like and imine-like adduct formation. 3-Ethyl-2-oxo-1,3-benzothiazoline-6-sulfonate can presumably witness a hydrazindyilidene-like adduct formation pathway, whereas 3-ethyl-2-imino-1,3-benzothiazoline-6-sulfonate testifies to the imine-like adduct formation pathway.
(5) The extent that the coupling reaction contributes to the reaction between antioxidants and ABTS^•+^ (e.g., kinetics and stoichiometry) is unclear due to the lack of quantitative estimation of their formation, and sometimes this may be quite considerable.

As for our opinion, undoubtedly there are questions in terms of the overall application of ABTS/PP assay, and this review adds to them, as the specifics for certain compound reactions, such as coupling, might bias the comparison of different antioxidants. Nevertheless, the ABTS-based assays can still be used with certain reservations, as was stated earlier, particularly for tracking changes in the same antioxidant system—for example, during storage and processing—or for composition effect evaluation. In addition, ABTS^•+^-mediated reactions can provide a perspective from other points of view, for instance, as a means for flavonoid analysis, and are, therefore, interesting to elucidate further.

## Figures and Tables

**Table 1:** The citation rates of the most widely used antioxidant activity/antioxidant capacity (AOA/AOC) methods and frequency of abbreviation usage. Data are from the Scopus database. ABTS: 2,2′-azino-bis(3-ethylbenzothiazoline-6-sulfonic acid), PP: potassium persulfate.

Method	References of Basic Publications	Citation Rate ^1^	Frequency of Abbreviation Use (Keywords Used)
TEAC	[1,2,16]	13,220	3772 ^2^ (“TEAC” or “Trolox equivalent antioxidant capacity”) since 1993
ABTS/PP decolorization assay	[1]	9845	10,109 (“ABTS antioxidant” or “ABTS antiradical” or “ABTS radical scavenging”) since 1988
DPPH	[17,18,19,20]	11,177	35,136 (“DPPH antioxidant” or “DPPH antiradical” or “DPPH radical scavenging”) since 1955
FRAP	[21,22,23]	11,040	9492 (“FRAP” or “Ferric reducing antioxidant power”) ^3^ since 1994
Folin–Ciocalteu	[24,25,26,27]	7630	2803 (“Folin–Ciocalteu antioxidant”) since 1976
ORAC	[28,29,30,31,32,33]	3478	3619 (“ORAC” or “Oxygen radical absorbance capacity”) since 1993
CUPRAC	[34,35,36]	1260	685 (“CUPRAC” or “Cupric ion reducing antioxidant capacity”) since 2004

^1^ From publication date to the end of 2018. ^2^ Possibly overestimated due to widespread usage of TEAC abbreviation for AOA/AOC results expression in different assays. ^3^ Words “rapamycin”, “photobleaching”, and “phosphatase” were excluded from this search to prevent overestimation due to other FRAP abbreviations not related to antioxidant activity assessment (i.e., fujimycin binding protein (FKBP)-rapamycin-associated protein, fluorescence recovery after photobleaching, and fluoride-resistant acid phosphatase).

**Table 2:** The comparison of ABTS^•+^ UV-VIS spectra characteristics.

λ(ABTS^•+^), nm	Extinction Coefficient, ε	Reference
415 nm	36,000 L∙mol^−1^∙cm^−1^ in water	[43,44]
414 nm	31,100 l mol^−1^ cm^−1^ in water (sodium phosphate buffer, pH 7.5) 33,630 L∙mol^−1^∙cm^−1^ in ethanol	[3,45]
734 nm	15,000 L∙mol^−1^∙cm^−1^ in water 16,000 L∙mol^−1^∙cm^−1^ in ethanol	[1]
730 nm	12,947 l mol^−1^ cm^−1^ in water (sodium phosphate buffer, pH 7.5) 14,750 L∙mol^−1^∙cm^−1^ in ethanol	[3,45]

**Table 3:** The comparison of the number of ABTS^•+^ molecules reduced by one molecule of antioxidant in decolorization and lag-time assays.

Antioxidant		The Calculated Number of ABTS^•+^ Molecules Reduced by One Molecule of Antioxidant ^1^	
Name	Formula	Decolorization Assay	Lag-Time Assay
Trolox	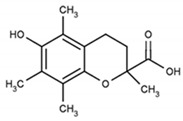	2.4	1.7
Quercetin	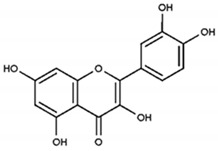	12.0	4.7
Morin	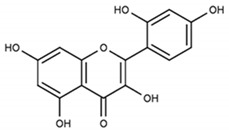	6.9	3.3
Rutin	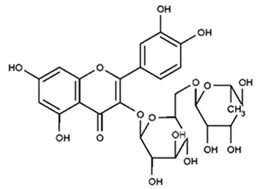	6.6	2.8
Taxifolin	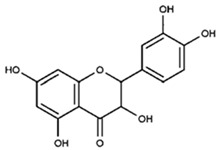	5.9	2.7
Apigenin	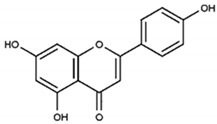	5.3	ND
Naringenin	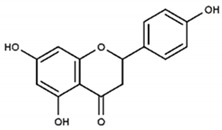	4.6	ND
Glutathione	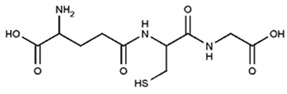	2.7	0.8
α-Tocopherol	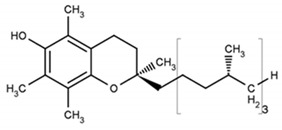	1.9	1.9

^1^ The calculated data from [58], considering ABTS^•+^ radical cation λ_max_ 734, ε 15,000 L mol^−1^ cm^−^^1^, and antioxidant concentrations that cause 50% inhibition or 15 min lag-time of the ABTS^•+^.

**Table 4:** Reagent concentrations in an ABTS/PP decolorization assay ^1^.

Antioxidant	Antioxidant Concentration Needed to Inhibit 50% of ABTS^•+^, µM	The Ratio Antioxidant/ABTS^•+^
Trolox	11.0	1:4
Glutathion	8.4	1:6
Taxifolin	4.1	1:11
Quercetin	1.8	1:26
α-Tocopherol	12.4	1:4

^1.^ Data from [58], the inhibited ABTS^•+^ radical cation concentration was calculated considering λ_max_ 734, ε 15,000 L mol*^−^*^1^ cm*^−^*^1^; the ratio antioxidant/ABTS^•+^ was calculated considering the initial concentration of ABTS^•+^ before antioxidant addition around 47 µM; the concentration of ABTS was around 31 µM; there was supposed to be no K_2_S_2_O_8_.

## Abbreviations

ABTS	2,2′-azino-bis(3-ethylbenzothiazoline-6-sulfonic acid) diammonium salt
AFMK	*N*1-acetyl-*N*2-formyl-5-methoxykynuramine
c3OHM	cyclic 3-hydroxymelatonin
CEP	concerted electron–proton transfer
COSY	correlation spectroscopy
CUPRAC	cupric reducing antioxidant capacity
DAD	diode-array detection
DPPH	2,2-diphenyl-1-picrylhydrazyl
ESI-HRMS	high resolution electrospray ionization mass spectrometry
ESI-MS	electrospray ionization mass spectrometry
ET-PT	electron transfer–proton transfer
FRAP	ferric reducing antioxidant power
FKBP	fujimycin binding protein
GBG	guaiacylglycerol-β-guaiacyl ether
GBGox	oxidation products of guaiacylglycerol-β-guaiacyl ether
GSH	glutathione
GSSG	glutathione disulfide
HAT	hydrogen atom transfer
HMBC	heteronuclear multiple bond correlation
HSQC	heteronuclear single quantum coherence
HPLC-ECD	high performance liquid chromatography, electrochemical detection
HPLC-UV	high performance liquid chromatography, ultraviolet detection
IC_50_	concentration which leads to 50% inhibition
IR	infrared spectroscopy
MS-MS	tandem mass spectrometry
NI	negative mode in electrospray ionization mass spectrometry
NMR	nuclear magnetic resonance
ORAC	oxygen radical absorbance capacity
PBS	phosphate buffer solution
PDA	photodiode array
PI	positive mode in electrospray ionization mass spectrometry
PP	potassium persulfate
QTOF-MS	quadrupole time of flight mass spectrometer
RP-UHPLC	reversed-phase ultrahigh-performance liquid chromatography
SET	single electron transfer
SIMS	secondary ion mass spectrometry
SPLET	sequential proton loss electron transfer
TEAC	trolox equivalent antioxidant capacity
TLC	thin layer chromatography
UV-VIS	ultraviolet–visible spectroscopy
